# Standard set of health outcome measures for older persons

**DOI:** 10.1186/s12877-017-0701-3

**Published:** 2018-02-02

**Authors:** Asangaedem Akpan, Charlotte Roberts, Karen Bandeen-Roche, Barbara Batty, Claudia Bausewein, Diane Bell, David Bramley, Julie Bynum, Ian D. Cameron, Liang-Kung Chen, Anne Ekdahl, Arnold Fertig, Tom Gentry, Marleen Harkes, Donna Haslehurst, Jonathon Hope, Diana Rodriguez Hurtado, Helen Lyndon, Joanne Lynn, Mike Martin, Ruthe Isden, Francesco Mattace Raso, Sheila Shaibu, Jenny Shand, Cathie Sherrington, Samir Sinha, Gill Turner, Nienke De Vries, George Jia-Chyi Yi, John Young, Jay Banerjee

**Affiliations:** 1 Department of Medicine for the Elderly, Aintree University Hospital NHS Foundation Trust, Lower Lane, Liverpool, L9 7AL UK; 2 International Consortium on Health Outcomes Measurement, London, UK; 3 International Consortium on Health Outcomes Measurement, Cambridge, USA; 4 Johns Hopkins Bloomberg School of Public Health, Johns Hopkins Older Americans Independence Center, Baltimore, USA; 5 Oxfordshire Clinical Commissioning Group, Oxford, UK; 6 LMU München, Munich University Hospital, Munich, Germany; 7 COBIC, London, UK; 8 NHS England, London, UK; 9 The Dartmouth Institute for Health Policy & Clinical Practice, Lebanon, USA; 10 John Walsh Centre for Rehabilitation Research, Sydney Medical School, University of Sydney, Sydney, Australia; 11 Ageing and Health Research Center, National Yang Ming University, Taipei, Taiwan; 12 Center for Geriatrics and Gerontology, Taipei Veterans General Hospital, Taipei, Taiwan; 13 Section of Clinical Geriatrics, Department of Neurobiology, Care Sciences and Society, Karolinska Institute, Stockholm, Sweden; 14 Cambridgeshire and Peterborough Clinical Commissioning Group, Cambridge, UK; 15 AgeUK, London, UK; 16 Havenziekenhuis, Rotterdam, Netherlands; 17 Older Person representative, Kingston, Canada; 18 Internal Medicine-Geriatrics, Faculty of Medicine, Universidad Peruana Cayetano Heredia, Lima, Peru; 19 Altarum Institute, Ann Arbor, USA; 20 University of Zurich, Zurich, Switzerland; 21 Erasmus University Medical Center, Rotterdam, Netherlands; 22 Sigma Theta Tau International Honor Society of Nursing, Indiana, USA; 23 UCL Partners, London, UK; 24 The George Institute for Global Health, University of Sydney, Sydney, Australia; 25 Departments of Medicine, Family and Community Medicine and the Institute of Health Policy Management and Evaluation, University of Toronto, Toronto, Canada; 26 Sinai Health System and University Health Network, Toronto, Canada; 27 British Geriatrics Society, London, UK; 28 University Nijmegen Medical Centre, Nijmegen, Netherlands; 29 Family caregiver, Taipei, Taiwan; 30 University of Leeds, Leeds, UK; 31 University Hospitals of Leicester NHS Trust, Leicester, UK; 32 NHS Digital, Leeds, UK

**Keywords:** Older people, Health outcomes

## Abstract

**Background:**

The International Consortium for Health Outcomes Measurement (ICHOM) was founded in 2012 to propose consensus-based measurement tools and documentation for different conditions and populations.This article describes how the ICHOM Older Person Working Group followed a consensus-driven modified Delphi technique to develop multiple global outcome measures in older persons.

The standard set of outcome measures developed by this group will support the ability of healthcare systems to improve their care pathways and quality of care. An additional benefit will be the opportunity to compare variations in outcomes which encourages and supports learning between different health care systems that drives quality improvement. These outcome measures were not developed for use in research. They are aimed at non researchers in healthcare provision and those who pay for these services.

**Methods:**

A modified Delphi technique utilising a value based healthcare framework was applied by an international panel to arrive at consensus decisions.To inform the panel meetings, information was sought from literature reviews, longitudinal ageing surveys and a focus group.

**Results:**

The outcome measures developed and recommended were participation in decision making, autonomy and control, mood and emotional health, loneliness and isolation, pain, activities of daily living, frailty, time spent in hospital, overall survival, carer burden, polypharmacy, falls and place of death mapped to a three tier value based healthcare framework.

**Conclusions:**

The first global health standard set of outcome measures in older persons has been developed to enable health care systems improve the quality of care provided to older persons.

**Electronic supplementary material:**

The online version of this article (10.1186/s12877-017-0701-3) contains supplementary material, which is available to authorized users.

## Background

The number of older people and their life expectancy has been rising steadily ranging from 50 years in resource poor to 83 years in resource rich regions [1]. Older people commonly have more than one chronic condition and have frequent encounters with healthcare providers [2]. Provision of care can be fragmented due to multiple assessments and treatments [3]. While focusing on a single condition may have advantages, a holistic approach with a review of outcomes that matter has greater value. Variation in outcomes of healthcare is a global challenge [4] and having the proposed set of outcome measures will facilitate and support reducing this variation.

Understanding what outcomes matter to patients would be valuable to clinicians and policymakers in aligning health care services to their needs. The aim of this project was to define a minimum set of outcomes for evaluating healthcare for older people. A Delphi technique was used to develop a balanced score card that was feasible to implement in routine clinical practice. An additional goal was to facilitate the creation of databases that can be compared and/or merged for analysis. This would support decision making being shared between providers, facilitate quality improvement and allow for benchmarking across organisations and countries.

The lack of outcome measurements that matter most to patients represents a barrier to health care improvement [5] and means providers have little information on which to judge the effectiveness of interventions. The ICHOM has to date developed 13 standard sets of outcome measures [6] and by 2017 at least 50% of the global disease burden will be covered. ICHOM (www.ICHOM.org) was founded in 2012 to promote value-based health care by defining global standard sets of outcome measures that matter to patients and promote adoption of these measures worldwide. This would be ICHOM’s first standard set of outcomes for a population as opposed to a specific condition such as cataracts, dementia or lung cancer [6].

ICHOM is a non-profit organisation supported by the Harvard Business School, Boston Consulting Group and the Karolinska Institute to transform health care systems worldwide by measuring and reporting patient outcomes in a standardised way. ICHOM organises global teams of physician leaders, outcomes researchers and patient advocates to define Standard Sets of outcomes per medical condition, and then drives adoption to enable health care providers globally to compare, learn, and improveA working group (WG) was organised by ICHOM, to represent a wide clinical, scientific and cultural background. Members (*n* = 31) included patient representatives, measurement experts, clinical, social and psychological researchers. Countries represented included Australia, Botswana, Canada, Germany, The Netherlands, Sweden, Switzerland, Taiwan, Peru, the United Kingdom, and the United States of America.

## Method

A modified Delphi technique was used to develop the standard set. The Delphi technique is an iterative, multistage process to actively transform opinion into group consensus [7]. Over a period of 10 months, the working group met eight times over teleconferences.

**Fig. 1:**
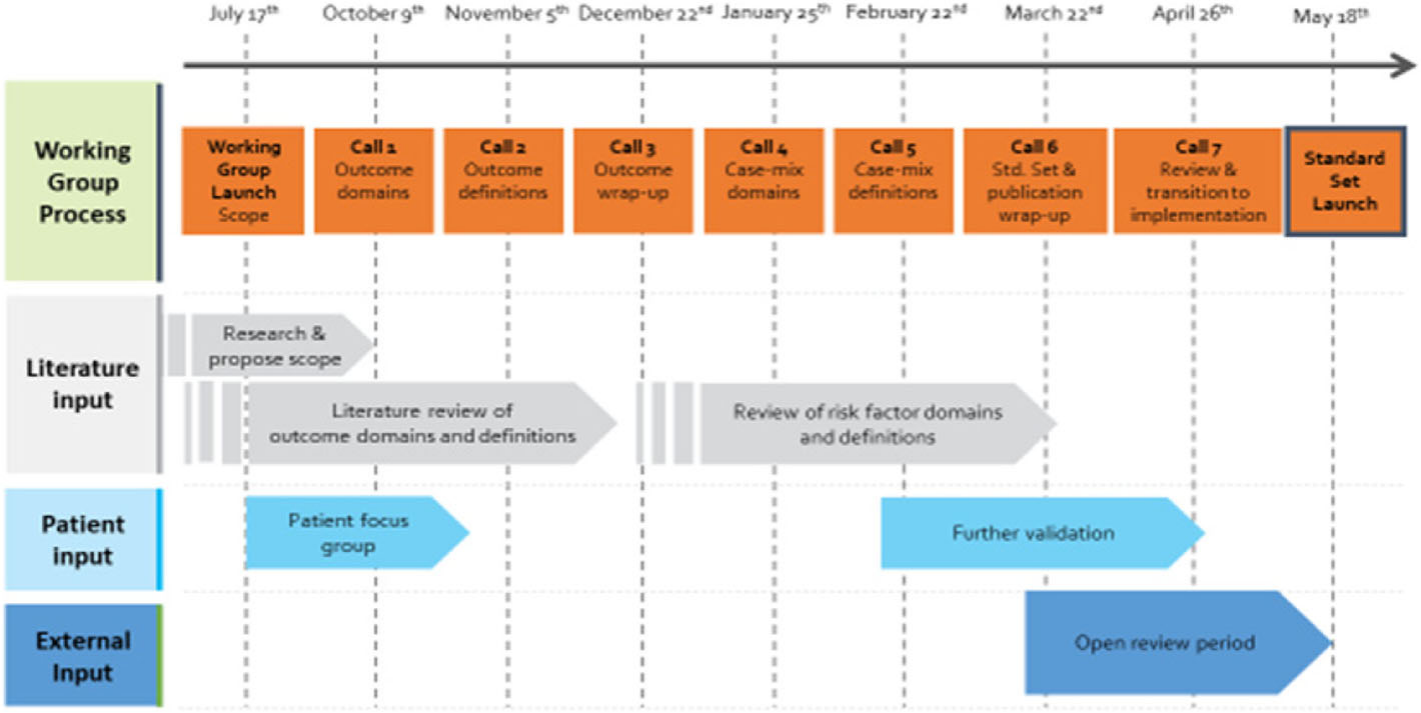
Showing the process and timeline for the working group. A flow chart showing the working group process

The goals and scope of the working group were discussed in the first teleconference. The second to fourth teleconferences (call 1 to 3 in Fig. 1) focused on the outcome domains and definitions to include in the standard set. In preparation for teleconferences 2–4, the working group were provided with information from literature reviews (Additional file 1: Table S1) and an older person’s and carer focus groups (Table 1). ICHOM organised an older people focus group with six attendees (age range 68–89) after the working group launch, to obtain their perspectives, using open-ended questions. Participants, consulted through Age UK’s networks, discussed which outcomes were of greatest importance to them. Age UK (http://www.ageuk.org.uk) is a charity dedicated to improving the lives of older people via a national network supported and facilitated by partnerships.

**Table 1:** Themes from the older persons and carer focus group

Amongst many discussed, the groups felt the following were most important:	However, there were a few new topics and points to consider:
• Social and community participation • Independence and remaining in own home • Quality of life and wellbeing • Avoiding inappropriate discharges and readmissions • Isolation • Loneliness and friendship • Physical disabilities – hearing, vision, continence, mobility • Hobbies and activities • Access to 24 h healthcare and social services • Avoiding falls • Delaying frailty • Care and respite for the carer • Malnutrition, weight loss and appetite • Physical symptom burden • Pain • Sleep quality	• Survival/mortality was seen as being less important than other outcomes – instead seen as inevitable and expected • Role in society e.g. formal/informal job or volunteering • Consistency of medical service/single coordinator of care

To support the decision making process the working group used a set of 4 criteria; represent the end results or ‘outcomes’ of care, represent what is important to OP and their families, feasible to capture and can be used for quality improvement programmes.

The discussion content was collated into online surveys. Working group members were asked to submit their feedback and votes via a web survey questionnaire. The survey had all the outcomes discussed with the level of agreement ranked during the teleconferences. Decisions resulting from the surveys required a minimum 50% of the working group membership participation. It was anticipated that due to time zone differences and schedules, this was a practical and reasonable standard to adopt given a fixed deadline by which the work had to be completed.

Teleconferences 5 and 6 (calls 4 and 5 Fig. 1) addressed case mix factors and definitions. Teleconferences 7 and 8 (calls 6 and 7 Fig. 1) focused on reviewing the agreed outcome domains, case mix factors and how the standard set would be shared with the healthcare community. Over the 10 months of the project, attendance for the teleconference meetings ranged between 51.7% to 75.9% (mean 61.1%). Three voting surveys were conducted with varying response rates. For a measure to be accepted as an outcome the working group set a standard of 70% and above of members voting to include a measure as an outcome.The final standard set was approved by all members of the working group.

PRISMA reporting principles were used as guidance for the literature search strategy [8]. Titles, keywords and abstracts were searched using MeSH or equivalent terms in the following databases PubMed/Medline, EMBASE, Psychinfo, Social Care online, Cumulative Index to Nursing and Allied Health Literature (CINAHL), COCHRANE, PsychInfo. Inclusion criteria included: (Aged, 80 and over OR Frail elderly or Comorbidity) AND (quality of life OR outcome assessment (healthcare) OR quality indicators), Paper and guidelines reporting on patient-reported and patient-centred outcomes, English language abstracts, reviews and randomised controlled trial,2005 onwards. Exclusion criteria included Non-English language, irretrievable, insufficient outcome data, unclear diagnoses, unvalidated outcomes.

Additional sources of information included existing measurement approaches adopted by longitudinal ageing surveys [9–38]. Figure 1 summarises the working group process.

Triangulating findings from the literature review and focus group with the working group discussions would strengthen the resultant outcome measures decided upon and highlight the key issues that most matter to older people. Experience of and satisfaction with care by older people and their carers including distress and mood was noted in quality of life literature reviews but did not come up specifically in the focus group discussions.

A three tiered hierarchy framework [39] has been utilised to categorise the outcome measures. Tier 1 is the health status achieved or retained with survival and then degree of recovery achieved. Tier 2 is the process of recovery with time to recovery and return to normal activities as well as the treatment burden such as side effects and complications.Tier 3 is sustainability of health with recurrences and long term consequences of care interventions.

A specific cut off age was considered inappropriate due to the range in life expectancies around the world. During the working group discussions, it was agreed that the last 10 years of life captured a period in which a person might be regarded as being old across the world and potentially seeking healthcare. Therefore, rather than specifying a fixed cut-off age as the inclusion population for this standard set, the working group recommended subtracting 10 years from the estimated life expectancy at 60 years in each country or region. The inclusion population would be those who are at or above this age. For example, in South Africa, the life expectancy at age 60 is 76 years old, therefore the inclusion population would be all those over the age of 66 [40–43]. These can be utilised for any society in the world where a particular age is viewed as old if it does not fall within the definition above. The principles that apply to older people would be the same. This respects and accepts that each society can define what old age is to them.

## Results

The suggested initial outcomes were chosen based on congruency across findings from the registries, surveys, literature searches and engagement with older people. A minority were chosen based on the consensus experience of the working group members. In the general category health status, quality of life, mortality, independence, remaining at home, carer health, and autonomy were deemed essential. In physical health, functional status, symptom occurrence, sleep, harm, frailty stage, nutrition, weight loss was also essential. Mental and psychological health had cognition, mood and loneliness as essential. Social network, support and isolation were essential in the social and community category. Length of stay, care coordination and discharge to place of choice were essential in healthcare utilisation. Dignity, shared decision making, access to information and advice were deemed essential under the experience/process category.

Tier 1 outcomes were overall survival, frailty and place of death. Tier 2 outcomes were polypharmacy, falls, participation in decision making and time spent in hospital. Tier 3 outcomes included loneliness and isolation, activities of daily living, pain, mood and emotional health, autonomy and control and carer burden. The results of the voting outcomes are summarised in Tables 2, 3, 4 and 5 summarises the outcome measures mapped to the tiers.

**Table 2:** Showing voting outcomes following round 1 survey of working group members. The % refers to the proportion of those who voted in support of each item

Round 1	Percent
Include	
Frailty	85
Overall health-related quality of life and wellbeing	100
Overall health status (self-reported)	96
Overall satisfaction with life (self-reported)	85
Physical functioning and disability (general)	88
General mobility	85
Social functioning	85
Carer quality of life and wellbeing	88
Carer depression	73
Cognitive functioning	100
Mental, psychological and emotional health	96
Independence	100
Ability to remain in own home	88
Carer health (general)	73
ADLs	96
Change in health status (self-reported)	88
Autonomy and control over daily life	100
Level of physical activity	81
Gait speed	81
Place of death	73
Place of death as preferred	73
Confusion/delirium	81
Isolation and loneliness	88
Mood	96
Anxiety	81
Overall burden of all other symptoms	77
Depression	81
Frequency of activity participation	73
Social/community engagement or participation	81
Confidence in ability to cope with own health problems	88
Experience of having been treated with dignity and respect	85
Confidence in role as participant in care	77
Other patient activation measures	73
Confidence in healthcare professionals	73
Hospital admissions	77
Hospital readmissions	85
Length of stay (hospital/rehab/nursing home/other)	77
Discharged to place of choice	77
Coordination of care	77
Inconclusive	
Functional mobility	58
Pain	58
Confidence in ability to access information and advice when needed	63
Confidence in ability to access appropriate healthcare	68
Feeling safe (generally)	68
Confidence in understanding of own health	58
Falls resulting in a fracture	58
Overall survival	68
Excluded	
Cause-specific survival	27
Blood pressure	15
Waist and hip circumference	8
Heart rate	15
Bone density	15
Lung function	12
Peak flow	8
Aortic calcification	12
Carotid intima-media thicknes	8
Standing and sitting height	12
Lean muscle mass and body composition	23
Condition-specific outcomes	15
Ability to work (formal/informal)	46
Dynamic balance	38
Static balance	38
Lower-limb strength	38
Grip strength	38
Oral health	42
Sleep quality	38
Weight loss	42
Appetite loss	42
Stiffness	27
Fatigue	46
Medication adherence	46

**Table 3:** Showing voting outcomes following round 2 survey of working group members. The % refers to the proportion of those who voted in support of each item

Round 2	Percent
Include	
Functional mobility	77
Pain	72
Falls resulting in a fracture	77
Inconclusive	
Confidence in ability to access information and advice when needed	50
Confidence in ability to access appropriate healthcare	64
Feeling safe (generally)	59
Confidence in understanding of own health	55
Overall survival	59
Overall burden of all physical symptoms	59
Continence	64
General experience of healthcare	55
Contact with healthcare (emergency service/doctor/nurse/outpatient clinic)	50
Pressure ulcers	50
Complications from treatment	59
Adverse medication effects	55
Falls resulting in seeking medical attention	59
Excluded	
Other palliative care specific outcomes	41
Relationships	68
Vision	45
Hearing	41
Sit to stand speed	36
Number of falls	68
Falls resulting in an admission to hospital	68

**Table 4:** Showing voting outcomes following round 3 survey of working group members. The % refers to the proportion of those who voted in support of each item

Round 3	Percent
Include	
Overall survival	71
Falls resulting in seeking medical attention	71
Polypharmacy (added in the third round)	75
Inconclusive	
Confidence in ability to access information and advice when needed	54
Confidence in ability to access appropriate healthcare	63
Confidence in understanding of own health	58
Complications from treatment	54
Excluded	
Feeling safe (generally)	42
Feeling safe within a healthcare organisation (added in the third round)	38
Overall burden of all physical symptoms	46
Continence	38
General experience of healthcare	29
Contact with healthcare (emergency service/doctor/nurse/outpatient clinic)	38
Pressure ulcers	46
Adverse medication effects	46

**Table 5:** Standard Set of Outcome Domains for Older People

Tiers	Outcome Domains	Supporting Information	Suggested Data Sources
Tier 1	Overall Survival	All cause survival	Administartive data
	Place of Death	Whether a preferred place to die has been expressed, the patient died in their usual place of residence and whether they died in their preferred place of death (if previously expressed)	Clinical data
	Frailty	Tracked via the Canadian Study on Health & Aging Clinical Frailty Scale	Clinical data
Tier 2	Polypharmacy^S190-191^	Includes the total number of prescribed medications, adverse drug events and whether medications make the patient unwell	Clinical data, Patient reported
	Falls^S192^	How many falls has the patient sustained in the last 12 months and how many falls have resulted in a fracture, need for any professional medical attention and hospitalization	Clinical data, Patient reported
	Particiapation in decision making	Includes confidence in; ability to cope with own health, role as participant in care (involved in discussions, planning) and healthcare professionals. Also includes the experience of having been treated with dignity and respect, coordination of care and discharge to place of choice	Patient reported
	Time spent in hospital	Number of hospital admissions, readmissions and total time spent in hospital over a year	Administrative data
Tier 3	Loneliness and isolation ^S193^	Tracked via the UCLA- 3-item scale	Patient reported
	Activities of daily living^S194-195^	Includes mobility and limitations to activities of daily living and tracked via the SF-36 and gait speed	Clinical data, Patient reported
	Pain^S196^	Tracked via the SF-36	Patient reported
	Mood and emotional health^S197^	Tracked via the SF-36	Patient reported
	Autonomy and control^S198^	How much control the patient has over their daily life tracked via the Adult Social Care Outcomes Toolkit	Patient reported
	Carer burden^S199^	Carer reported burden tracked via the 4-item screening Zarit Burden Interview	Carer reported

Key to Table 5

*UCLA* University of California, Los Angeles -3 Item Scale [59]

*SF36* Short Form (36) Health Survey [60]

*ASCOT* Adult Social Care Outcomes Toolkit [61]

*ZBI* Zarit Burden Interview [62]

*CSHACFS* Canadian Study of Health and Ageing Clinical Frailty Scale [63]

Polypharmacy

S190. Tjia J, Velten SJ, Parsons C et al. Studies to reduce unnecessary medication use in frail older adults: a systematic review. Drugs Aging 2013;30(5):285-307

S191. Shrank WH, Polinski JM, Avorn J. Quality indicators for medication use in vulnerable elders. J Am Geriatr Soc 2007;55 Suppl 2: S373-82

Falls

S192. Chang JT, Ganz DA. Quality indicators for falls and mobility problems in vulnerable elders. J Am Geriatr Soc 2007;55 Suppl 2: S327-34

Loneliness and isolation

S193. Hughes ME, Waite LJ, Hawkley LC et al. A short scale for measuring loneliness in large surveys: Results from two population-based studies. Res Aging 2004;26(6):655–672

Activities of daily living

S194. 36-Item Short Form Survey (SF-36). Available at http://www.rand.org/health/surveys_tools/mos/36-item-short-form.html Accessed on the 13 November 2016

S7195. Peel NM, Kuys SS, Klein K. Gait speed as a measure in geriatric assessment in clinical settings: a systematic review. J Gerontol A Biol Sci Med Sci 2013;68(1):39-46

Pain

S196. 36-Item Short Form Survey (SF-36). Available at http://www.rand.org/health/surveys_tools/mos/36-item-short-form.html Accessed on the 13 November 2016

Mood and emotional health

S197. 36-Item Short Form Survey (SF-36). Available at: http://www.rand.org/health/surveys_tools/mos/36-item-short-form.html Accessed on the 13 November 2016

Autonomy and control

S198. Available at: http://www.pssru40.org.uk/ascot Accessed on the 13 November 2016

Carer burden

S199. Bedard M, Molloy DW, Squire L et al. The Zarit Burden Interview: a new short version and screening version. Gerontologist 2001;41: 652-657

The collection of a minimum set of baseline characteristics is recommended to allow case-mix adjustments [44, 45] Case-mix adjustment is a useful and fair way for making comparisons among health care providers. Taking these into consideration reduces disadvantages in comparative ratings due to differences in the underlying population of interest.

The working group agreed:

- Demographic factors: Such as age, gender, level of education, living arrangements, marital status and ethnicity. Items are harmonised to other ICHOM surveys. The educational level should be assessed following the International Standard Classification of Education [46] to allow global comparisons.
- Condition specific variables: These were frailty stage, type of medication used, total number of medications and baseline cognition.
- Systemic variables: Included were co-morbidities, smoking, alcohol use, weight, height, body mass index, vision and hearing impairment, and baseline activities of daily living.

A reference guide is freely available online that further describes the recommended instruments, data sources and provides detailed information (www.ichom.org).

## Discussion

A standard set of outcome measures that matter to older people has been developed by a global panel of interdisciplinary professionals,older people and their carers.

The strengths of this project include the global interdisciplinary collaboration, involving older people and their carers and triangulating findings from a focus group, professional experience and the published literature. Obtaining information from various sources was important as not surprisingly not all domains were articulated in the single focus group due to its small sample. This also focused on a subset of a population rather than on a specific medical condition. To date no other set of outcome measures for older people has been developed using this approach. This approach has reduced the chances of excluding important themes that matter to older people. In attempting to be comprehensive and for the findings to be feasible for implementation, some themes had to be excluded. This does not mean they are not important but feasibility of the outcomes being used was regarded by the working group to be critical. The outcome measures have not been developed for use by academic researchers and will therefore not meet criteria for use by that group. The measures have been specifically developed for practical use by healthcare providers and those who pay for these services.

The framework utilised to develop these outcomes is based on Porter’s outcome hierarchy [39]. Tier 1 is the most important with the outcome being survival or the best possible state achieved for a condition. Tier 2 outcomes are the issues related to achieving tier 1 outcomes such as the time to recovery from a flare up of a chronic disease or recovery from an acute disease. Included in this tier 2 are all the harms associated with investigations and treatment. Tier 3 outcomes relate to long term health status.

Healthcare providers should appreciate and understand the perception, attitude and behaviour of those they care for [47]. In this context, “what matters to you” as a recipient of healthcare is more important than “what is the matter with you.” We have attempted to balance the information derived from previous studies to compensate for this by incorporating the views of OP and their carers. We hope that whilst not ideal, concerted efforts were made to ensure that the voice of OP and their carers were incorporated.

The value of performance based measures including grip strength as health outcomes for older adults [48] was discussed. The evidence base supporting the value of such measures for providing integrative assessments of older persons’ health, and for identifying persons at risk of a decline in health was recognized. The majority of the group considered the collection of such measures burdensome as part of a minimum set of indicators to be included in the standard set but endorse the value of incorporating them in specialty geriatric settings.

Frailty is well recognised [49, 50]. For providers, understanding the proportion of those becoming frail will aid their future resource allocation, service planning and prevention strategies [51, 52]. There was agreement for a frailty measure as a risk factor for outcome measure adjustment but much less agreement concerning the role of a frailty measure as a service outcome. Indeed, this was the most discussed topic.While the phenotype model [53] remains the gold standard for diagnosing frailty, the cumulative deficit model [54] was viewed by a majority as what clinicians will identify with more easily. Both have been validated in aiding clinical decision making [48, 55] and [56]. The Canadian Study of Health and Ageing (CSHA) Clinical Frailty Scale [43] was recommended as the tool to be used in the standard set to assess frailty. It mirrors clinical judgement, is objective [57] and can be used in places with no electronic health records. However, alternative frailty tools may become widely implemented in some countries. For example, an electronic frailty index is now available for use for over 90% of general practitioners in England [58] (http://ageing.oxfordjournals.org/content/early/2016/03/03/ageing.afw039.full) and*,* an online tool (www.johnshopkinssolutions.com/solution/frailty) is available for frailty assessment utilising the phenotype model.

At first glance, polypharmacy, falls and length of stay in hospital may not appear to be outcome measures. This is where triangulation of findings from focus group and the working group discussions added value to this project. These three areas were things that mattered to older people, their carers and clinicians. It was felt that without keeping track of these in the form of outcome measures it could easily fall off the radar of health systems caring for older people. The SF-36 and other tools to capture the metrics around the outcome measures were chosen solely for very practical reasons. It had to be free to use and cover as many of the outcome measures to reduce the number of tools and complexity of use associated with this.

The final set of outcome measures arrived at has been reduced down from the original set at the outset of the project. In settling for a cut off, the working group applied feasibility and comprehensiveness as a guiding principle. In using such a diverse group, it is hoped that a reasonable balance has been struck.

The working group consensus was to measure the standard set outcomes longitudinally over time. A minimum annual frequency was recommended given the challenges of measurement and capturing population level changes. It was acknowledged that while some stakeholders might be interested and keen to collect these data more frequently and / or at each healthcare encounter, to recommend more than an annual collection could be too prescriptive and burdensome for providers.

This was an ambitious project and the working group recognised that it was unlikely to satisfy everyone. This is however a good starting point and further outcome measures should be explored and developed for specific niche groups such as older people with frailty, cognitive impairment, physical disability as well as exploring outcome measures that would be relevant for carers and researchers in old age health. Furthermore as these outcome measures start being used, areas for improveing them would arise and allow for them to be amended continuously to make them relevant and fit for purpose as our healthcare environment continues to change.

## Conclusion

Through the efforts reported in this paper, the ICHOM older people working group defined a standard set of recommended outcome measures that matter to older people. This is a first effort towards a standardisation of outcome measures to improve the quality of care for older people. Much further work remains to be done but in the meantime, itwould be ideal for national data sets to include information which allows these outcomes to be derived routinely.

## Additional file

Additional file 1: All the references cited in the **Tables S1**. (DOCX 72 kb)

## Abbreviations

ASCOT: Adult social care outcomes toolkit
CSHACFS: Canadian study of health and ageing clinical frailty scale
ICHOM: International consortium for health outcomes measurement
OP: Older people
SF36: Short form (36) health survey
UCLA: University of California, Los Angeles
WG: Working Group
ZBI: Zarit burden interview
