# Supplementary material for: Standard set of health outcome measures for older persons
Source: BMC Geriatr. 2018 Feb 2;18:36. doi: 10.1186/s12877-017-0701-3 (PMC5797357; doi:10.1186/s12877-017-0701-3)
Supplement: Additional file 1: — All the references cited in the Tables S1. (DOCX 72 kb) [file 12877_2017_701_MOESM1_ESM.docx]

Additional file 1

Below are references and links to further information and reading on topics listed in Tables S1 and S6 in the main article.

**Table S1**

Table S1 Themes retrieved from articles reviewed

| **Categories** | **Outcome domains** | **Total # of references** |
| --- | --- | --- |
| **General** | Remaining in own home (avoiding institutions)^S1-S13^ | 13 |
|  | Quality of life^S14-S43^ | 30 |
|  | Receiving care in desired/usual place of residence (or avoiding hospital admissions)^S44-S58^ | 15 |
|  | Mortality/survival^S59-S63^ | 5 |
| **Physical health** | Functional independence with ADLs^S64-S92^ | 29 |
|  | Mobility^S93-S122^ | 30 |
|  | Falls^S123-S132^ | 10 |
|  | Minimising number of medications prescribed^S133-S136^ | 4 |
|  | Good palliative care^S137-S143^ | 7 |
|  | Good vision^S144^ | 1 |
|  | Continence^S145-S146^ | 2 |
|  | Good nutritional status^S147-S153^ | 7 |
|  | Pain^S154-S156^ | 3 |
| **Mental and psychological health** | Maintaining cognition^S157-S163^ | 7 |
| **Experience measures** | Treated with respect and dignity^S164-S168^ | 5 |
|  | Involved in discussions and decisions^S169-S179^ | 11 |
|  | Reduced anxiety and worry about healthcare^S180-S189^ | 10 |

General

Remaining in own home (avoiding institutions)

1. Sandberg M, Kristensson J, Midlöv P et al. Effects on healthcare utilization of case management for frail older people: a randomized controlled trial (RCT). Arch Gerontol Geriatr 2015;60(1):71-81
2. Senior HE, Parsons M, Kerse N et al. Promoting independence in frail older people: a randomised controlled trial of a restorative care service in New Zealand. Age Ageing 2014 ;43(3):418-24
3. Ruikes FG, Meys AR, van de Wetering G et al. The CareWell-primary care program: design of a cluster controlled trial and process evaluation of a complex intervention targeting community-dwelling frail elderly. BMC Fam Pract 2012; 13:115
4. Partridge JS, Harari D, Dhesi JK. Frailty in the older surgical patient: a review. Age Ageing 2012;41(2):142-7
5. Ollonqvist K, Aaltonen T, Karppi SL et al. Network-based rehabilitation increases formal support of frail elderly home-dwelling persons in Finland: randomised controlled trial. Health Soc Care Community 2008;16(2):115-25
6. Gnanadesigan N, Fung CH. Quality indicators for screening and prevention in vulnerable elders. J Am Geriatr Soc 2007;55 Suppl 2: S417-23
7. Kircher TT, Wormstall H, Müller PH et al. A randomised trial of a geriatric evaluation and management consultation services in frail hospitalised patients. Age Ageing 2007;36(1):36-42
8. Flood C, Mugford M, Stewart S et al. Occupational therapy compared with social work assessment for older people. An economic evaluation alongside the CAMELOT randomised controlled trial. Age Ageing 2005;34(1):47-52
9. Stewart S, Harvey I, Poland F et al. Are occupational therapists more effective than social workers when assessing frail older people? Results of CAMELOT, a randomised controlled trial. Age Ageing 2005 Jan;34(1):41-6
10. Roe, B, Beech R, Harris M et al. Improving quality of life for older people in the community: findings from a local Partnerships for Older People Project innovation and evaluation. Prim Health Care Res Dev 2011;12(3), 200-213
11. Zekry D, Loures VB, Graf C et al. Prospective Comparison of 6 Comorbidity Indices as Predictors of 1-Year Post-Hospital Discharge Institutionalization, Readmission, and Mortality in Elderly Individuals, Journal Of the American Medical Directors Association 2012;13:3:272-278
12. Drubbel I, de Wit NJ, Bleijenberg N et al. Prediction of adverse health outcomes in older people using a frailty index based on routine primary care data. The journals of gerontology. Series A, Biological sciences and medical sciences 2013;68:301-308
13. Kendrick D, Kumar A, Carpenter H et al. Exercise for reducing fear of falling in older people living in the community. Cochrane Database of Systematic Reviews 2014;11:CD009848.

Quality of life

1. Aspden T, Bradshaw SA, Playford ED et al. Quality-of-life measures for use within care homes: a systematic review of their measurement properties. Age and Ageing 2014;43: 596-603
2. Bowling, Ann. Quality of life: measures and meanings in social care research. London: NIHR School for Social Care Research 2014.
3. Centre for Policy on Ageing. Outcomes against which the success of prevention should be monitored. Centre for Policy on Ageing. Available at: <http://www.cpa.org.uk/information/reviews/CPA-Rapid-Review-Outcomes-against-which-the-success-of-prevention-should-be-monitored.pdf> Accessed on the 28 November 2016
4. Cowen R. Ageing well in Dorset: how not to be invisible. Journal of Integrated Care 2009;17: 31-36
5. Gethin-Jones, S. Outcomes and well-being part 1: a comparative longitudinal study of two models of homecare delivery and their impact upon the older person self-reported subjective well-being. Working with Older People 2012;16: 22-30
6. Hartgerink JM, Cramm J, van Wijngaarden J et al. A framework for understanding outcomes of integrated care programs for the hospitalised elderly. International Journal of Integrated Care 2013;13(4)
7. Hurtley R, Duff P. Achieving excellence in person-centred living. Nursing and Residential Care 2012;14: 99-102
8. King AI, Parsons M, Robinson E et al. Assessing the impact of a restorative home care service in New Zealand: a cluster randomised controlled trial. Health Soc Care Community, 2012;20(4):365-374
9. Manthorpe J. Long-term impact of home care reablement. Community Care 201;32-33
10. Netten A, Darton R, Bäumker T et al. Improving housing with care choices for older people: an evaluation of extra care housing. London: Housing Learning and Improvement Network; Personal Social Services Research Unit 2011. Available at: <http://www.housinglin.org.uk/_library/Resources/Housing/Research_evaluation/PSSRUsummary.pdf> Accessed on the 27 November 2016
11. Netten A, Beadle-Brown J, Trukeschitz B et al. Measuring the outcomes of care homes: final report. Canterbury: Personal Social Services Research Unit.

Available at: <http://www.pssru.ac.uk/pdf/dp2696_2.pdf> Accessed on the 27 November 2016

1. Prior MK, Bahret BA, Pasupuleti S. The efficacy of a senior outreach program in the reduction of hospital readmissions and emergency department visits among chronically ill seniors. Social Work in Health Care 2012; 51: 345-360
2. Social Care Institute for Excellence. Improving care packages and outcomes of older people. Community Care 2008;32-33
3. Towers C. Evaluation toolkit for providers: an assessment tool and action plan for organisations to improve the quality of life for people with learning disabilities as they grow older. Available at: <http://arcuk.org.uk/realchangechallenges/files/2014/03/Growing-older-evaluation-toolkit_interactive.pdf> Accessed on the 28 November 2016
4. Croucher K. Making the case for retirement villages. Joseph Rowntree Foundation. Available at: <https://www.jrf.org.uk/report/making-case-retirement-villages>. Accessed on the 28 November 2016
5. Chung E, Tse V, Chan L. The use of botulinum toxin a in refractory non-neurogenic overactive bladder in the elderly: A prospective review of intermediate term outcome with quality of life outcome assessment. Neurourology and Urodynamics 2012; 31:980.
6. Frisoli A, Ingham SJM, Paes TT et al. Frailty predictors and outcomes among older patients with cardiovascular disease: Data from Fragicor. Archives of Gerontology and Geriatrics 2015; 61:1-7
7. Reimer T, Gerber B. Quality-of-life considerations in the treatment of early-stage breast cancer in the elderly. Drugs & Aging 2010; 27,10:791-800
8. van Leeuwen K, Bosmans J, Jansen A et al. Comparing measurement properties of the EQ-5D-3L, ICECAP-O, and ASCOT in frail older adults. Value in Health 2015; 18:1:35-43
9. Masel M, Ostir, G, Ottenbacher K. Frailty, mortality, and health-related quality of life in older Mexican Americans, Journal of The American Geriatrics Society 2010;58: 11: 2149-2153
10. Dechamps A, Onifade C, Decamps A et al. Health-related quality of life in frail institutionalized elderly: Effects of a cognition-action intervention and Tai Chi, Journal of Aging and Physical Activity 2009;17: 2:236-248
11. Böckerman P, Johansson E, Saarni S. Institutionalisation and subjective wellbeing for old-age individuals: Is life miserable in care homes?, Ageing & Society 2012;32:7: 1176-1192
12. Andersson M, Hallberg I, Edberg A. Old people receiving municipal care, their experiences of what constitutes a good life in the last phase of life: A qualitative study, International Journal of Nursing Studies 2008;45:6:818-828
13. Coleman S, Cunningham C, Walsh J et al. Outcomes among older people in a post-acute inpatient rehabilitation unit, Disability and Rehabilitation: An International Multidisciplinary Journal 2012;34:15:1333-1338
14. Gregersen M, Jordansen M, Gerritsen D. Overall Quality of Life (OQoL) questionnaire in frail elderly: A study of reproducibility and responsiveness of the Depression List (DL), Archives of Gerontology and Geriatrics 2015;60:1:22-27
15. Hjaltadóttir I, Gústafsdóttir M. Quality of life in nursing homes: Perception of physically frail elderly residents, Scandinavian Journal of Caring Sciences 2007;21:1:48-55
16. Reimer T, Gerber B. Quality-of-life considerations in the treatment of early-stage breast cancer in the elderly, Drugs & Aging 2010;27:10:791-800
17. Groninger H, Childress M. Samuel Beckett's 'Rockaby': Dramatizing the plight of the solitary elderly at life's end, Perspectives in Biology and Medicine 2007;50:2:260-275
18. Lim J, Kim G, Kim E et al. The effects of community-based visiting care on the quality of life, Western Journal of Nursing Research 2013;35:10: 1280-1291
19. Sato D, Kaneda K, Wakabayashi H et al. The water exercise improves health-related quality of life of frail elderly people at day service facility, Quality of Life Research: An International Journal of Quality of Life Aspects of Treatment, Care & Rehabilitation 2007;16:10:1577-1585

Receiving care in desired/usual place of residence (or avoiding hospital admissions)

1. 1.Sandberg M, Kristensson J, Midlöv P et al. Effects on healthcare utilization of case management for frail older people: a randomized controlled trial (RCT). Arch Gerontol Geriatr 2015;60(1):71-81
2. Ruikes FG, Meys AR, van de Wetering G et al. The CareWell-primary care program: design of a cluster controlled trial and process evaluation of a complex intervention targeting community-dwelling frail elderly. BMC Fam Pract 2012; 13:115
3. Abernethy AP, Currow DC, Shelby-James T et al. Delivery strategies to optimize resource utilization and performance status for patients with advanced life-limiting illness: results from the "palliative care trial" [ISRCTN 81117481]. J Pain Symptom Manage 2013 ;45(3):488-505
4. van Hout HP, Jansen AP, van Marwijk HW et al. Prevention of adverse health trajectories in a vulnerable elderly population through nurse home visits: a randomized controlled trial [ISRCTN05358495]. J Gerontol A Biol Sci Med Sci 2010;65(7):734-42
5. Eklund K, Wilhelmson K. Outcomes of coordinated and integrated interventions targeting frail elderly people: a systematic review of randomised controlled trials. Health Soc Care Community 2009;17(5):447-58
6. Hopp F, Woodbridge P, Subramanian U et al. Outcomes associated with a home care telehealth intervention. Telemed J E Health 2006;12(3):297-307
7. Flood C, Mugford M, Stewart S et al. Occupational therapy compared with social work assessment for older people. An economic evaluation alongside the CAMELOT randomised controlled trial. Age Ageing 2005;34(1):47-52
8. Stewart S, Harvey I, Poland F et al. Are occupational therapists more effective than social workers when assessing frail older people? Results of CAMELOT, a randomised controlled trial. Age Ageing 2005;34(1):41-6
9. Prior MK, Bahret BA, Pasupuleti S. The efficacy of a senior outreach program in the reduction of hospital readmissions and emergency department visits among chronically ill seniors. Social Work in Health Care 2012; 51: 345-360
10. Chow S, Wong F. A randomized controlled trial of a nurse-led case management programme for hospital-discharged older adults with co-morbidities, Journal of Advanced Nursing 2014;70:10:2257-2271
11. Zekry D, Loures VB, Graf C et al. Prospective Comparison of 6 Comorbidity Indices as Predictors of 1-Year Post-Hospital Discharge Institutionalization, Readmission, and Mortality in Elderly Individuals, Journal of The American Medical Directors Association 2012;13:3:272-278
12. Kergoat M, Latour J, Lebel P et al. Quality-of-Care Processes in Geriatric Assessment Units: Principles, Practice, and Outcomes, Journal of The American Medical Directors Association 2012;13: 5:459-463
13. Drubbel I, de Wit N.J, Bleijenberg N et al. Prediction of adverse health outcomes in older people using a frailty index based on routine primary care data. The journals of gerontology. Series A, Biological sciences and medical sciences 2013;68:301-308
14. Eklund K, Wilhelmson K. Outcomes of coordinated and integrated interventions targeting frail elderly people: A systematic review of randomised controlled trials. Health and Social Care in the Community 2009; 17:447-458
15. Kendrick D, Kumar A, Carpenter H et al. Exercise for reducing fear of falling in older people living in the community. Cochrane Database of Systematic Reviews 2014;11: CD009848

Mortality/survival

1. 1.Drubbel I, de Wit N.J, Bleijenberg N et al. Prediction of adverse health outcomes in older people using a frailty index based on routine primary care data. The journals of gerontology. Series A, Biological sciences and medical sciences 2013;68:301-308
2. Frigerio M, Bruschi G, Klugmann S. Letter by Frigerio et al Regarding Article, "long-Term Outcomes of Inoperable Patients with Aortic Stenosis Randomly Assigned to Transcatheter Aortic Valve Replacement or Standard Therapy" Circulation 2015;132: e117
3. Guzik AK, Raman R, Ernstrom K et al. Iv rt-PA treatment response of the stroke100 club: Systematic technique for risk and outcome measurements using key elements totalling 100. Stroke 2013;44
4. Merli F, Luminari S, Rossi G et al. Outcome of frail elderly patients with diffuse large B-cell lymphoma prospectively identified by Comprehensive Geriatric Assessment: Results from a study of the Fondazione Italiana Linfomi. Leukemia and Lymphoma 2014; 55:38-43
5. Gutiérrez F, Masiá M. Improving outcomes of elderly patients with community-acquired pneumonia, Drugs & Aging 2008;25:7:586-610

Physical health

Functional independence with ADLs

1. O'Caoimh R, Gao Y, Svendrovski A et al. Screening for markers of frailty and perceived risk of adverse outcomes using the Risk Instrument for Screening in the Community (RISC). BMC Geriatr 2014; 19:14:104
2. Sepehri A, Beggs T, Hassan A et al. The impact of frailty on outcomes after cardiac surgery: a systematic review. J Thorac Cardiovasc Surg. 2014;148(6):3110-7
3. Beaupre LA, Binder EF, Cameron ID et al. Maximising functional recovery following hip fracture in frail seniors. Best Pract Res Clin Rheumatol 2013;27(6):771-88
4. Senior HE, Parsons M, Kerse N et al. Promoting independence in frail older people: a randomised controlled trial of a restorative care service in New Zealand. Age Ageing 2014 ;43(3):418-24
5. Cadore EL, Casas-Herrero A, Zambom-Ferraresi F et al. Multicomponent exercises including muscle power training enhance muscle mass, power output, and functional outcomes in institutionalized frail nonagenarians. Age (Dordr) 2014;36(2):773-85
6. Eklund K, Wilhelmson K, Gustafsson H et al. One-year outcome of frailty indicators and activities of daily living following the randomised controlled trial: "Continuum of care for frail older people". BMC Geriatr 2013; 22:13:76
7. Giné-Garriga M, Guerra M, Unnithan VB. The effect of functional circuit training on self-reported fear of falling and health status in a group of physically frail older individuals: a randomized controlled trial. Aging Clin Exp Res 2013;25(3):329-36
8. Cameron ID, Fairhall N, Langron C et al. A multifactorial interdisciplinary intervention reduces frailty in older people: randomized trial. BMC Med 2013; 11:65
9. Ruikes FG, Meys AR, van de Wetering G et al. The CareWell-primary care program: design of a cluster controlled trial and process evaluation of a complex intervention targeting community-dwelling frail elderly. BMC Fam Pract 2012; 13:115
10. Abernethy AP, Currow DC, Shelby-James T et al. Delivery strategies to optimize resource utilization and performance status for patients with advanced life-limiting illness: results from the "palliative care trial" [ISRCTN 81117481]. J Pain Symptom Manage 2013 ;45(3) :488-505
11. van Mourik Y, Moons KG, Bertens LC et al. Triage of frail elderly with reduced exercise tolerance in primary care (TREE). A clustered randomized diagnostic study. BMC Public Health 2012; 12:385
12. Chou CH, Hwang CL, Wu YT. Effect of exercise on physical function, daily living activities, and quality of life in the frail older adults: a meta-analysis. Arch Phys Med Rehabil 2012;93(2):237-44
13. de Vries NM, van Ravensberg CD, Hobbelen JS et al. Effects of physical exercise therapy on mobility, physical functioning, physical activity and quality of life in community-dwelling older adults with impaired mobility, physical disability and/or multi-morbidity: a meta-analysis. Ageing Res Rev 2012;11(1):136-49
14. Szturm T, Betker AL, Moussavi Z et al. Effects of an interactive computer game exercise regimen on balance impairment in frail community-dwelling older adults: a randomized controlled trial. Phys Ther 2011;91(10):1449-62
15. Wilhelmson K, Duner A, Eklund K et al. Design of a randomized controlled study of a multi-professional and multidimensional intervention targeting frail elderly people. BMC Geriatr 2011; 11:24
16. Weening-Dijksterhuis E, de Greef MH, Scherder EJ et al. Frail institutionalized older persons: A comprehensive review on physical exercise, physical fitness, activities of daily living, and quality-of-life. Am J Phys Med Rehabil 2011;90(2):156-68
17. Metzelthin SF, van Rossum E, de Witte LP et al. The reduction of disability in community-dwelling frail older people: design of a two-arm cluster randomized controlled trial. BMC Public Health 2010; 10:511
18. Rydwik E, Frändin K, Akner G. Effects of a physical training and nutritional intervention program in frail elderly people regarding habitual physical activity level and activities of daily living--a randomized controlled pilot study. Arch Gerontol Geriatr 2010;51(3):283-9
19. Sato D, Kaneda K, Wakabayashi H et al. Comparison two-year effects of once-weekly and twice-weekly water exercise on health-related quality of life of community-dwelling frail elderly people at a day-service facility. Disabil Rehabil 2009;31(2):84-93
20. Ollonqvist K, Aaltonen T, Karppi SL et al. Network-based rehabilitation increases formal support of frail elderly home-dwelling persons in Finland: randomised controlled trial. Health Soc Care Community 2008;16(2):115-25
21. de Rooij SE, Abu-Hanna A, Levi M et al. Factors that predict outcome of intensive care treatment in very elderly patients: a review. Crit Care 20059(4) : R307-14
22. Groll DL, To T, Bombardier C et al. The development of a comorbidity index with physical function as the outcome. J Clin Epidemiol 2005;58(6):595-602
23. Flood C, Mugford M, Stewart S et al. Occupational therapy compared with social work assessment for older people. An economic evaluation alongside the CAMELOT randomised controlled trial. Age Ageing 2005;34(1):47-52
24. Stewart S, Harvey I, Poland F et al. Are occupational therapists more effective than social workers when assessing frail older people? Results of CAMELOT, a randomised controlled trial. Age Ageing 2005;34(1):41-6
25. Manthorpe J. Long-term impact of home care reablement. Community Care 2011;32-33
26. Netten A, Darton R, Bäumker T et al. Improving housing with care choices for older people: an evaluation of extra care housing. London: Housing Learning and Improvement Network; Personal Social Services Research Unit 2011. Available at: <http://www.housinglin.org.uk/_library/Resources/Housing/Research_evaluation/PSSRUsummary.pdf> Accessed on the 27 November 2016
27. Tinetti M, McAvay G, Chang S et al. Contribution of Multiple Chronic Conditions to Universal Health Outcomes... [corrected] [published erratum appears in J Am Geriatr Soc 2011; 59:2196], Journal of The American Geriatrics Society 2011;59:9:1686-1691
28. Coleman S, Cunningham C, Walsh J et al. Outcomes among older people in a post-acute inpatient rehabilitation unit, Disability and Rehabilitation: An International Multidisciplinary Journal 2012; 34:15:1333-1338
29. Sato D, Kaneda K, Wakabayashi H et al. The water exercise improves health-related quality of life of frail elderly people at day service facility, Quality of Life Research: An International Journal of Quality of Life Aspects of Treatment, Care & Rehabilitation 2007;16:10:1577-1585

Mobility

1. Jeon MY, Jeong H, Petrofsky J et al. Effects of a randomized controlled recurrent fall prevention program on risk factors for falls in frail elderly living at home in rural communities. Med Sci Monit 2014; 20:2283-91
2. Sepehri A, Beggs T, Hassan A et al. The impact of frailty on outcomes after cardiac surgery: a systematic review. J Thorac Cardiovasc Surg 2014;148(6):3110-7
3. Timmer AJ, Unsworth CA, Taylor NF. Rehabilitation interventions with deconditioned older adults following an acute hospital admission: a systematic review. Clin Rehabil 2014;28(11):1078-86
4. Beaupre LA, Binder EF, Cameron ID et al. Maximising functional recovery following hip fracture in frail seniors. Best Pract Res Clin Rheumatol 2013;27(6):771-88
5. Senior HE, Parsons M, Kerse N et al. Promoting independence in frail older people: a randomised controlled trial of a restorative care service in New Zealand. Age Ageing 2014 ;43(3):418-24
6. Cadore EL, Casas-Herrero A, Zambom-Ferraresi F et al. Multicomponent exercises including muscle power training enhance muscle mass, power output, and functional outcomes in institutionalized frail nonagenarians. Age (Dordr) 2014;36(2):773-85
7. Giné-Garriga M, Guerra M, Unnithan VB. The effect of functional circuit training on self-reported fear of falling and health status in a group of physically frail older individuals: a randomized controlled trial. Aging Clin Exp Res 2013;25(3):329-36
8. Cameron ID, Fairhall N, Langron C et al. A multifactorial interdisciplinary intervention reduces frailty in older people: randomized trial. BMC Med 2013; 11:65
9. Mentz RJ, Schulte PJ, Fleg JL et al. Clinical characteristics, response to exercise training, and outcomes in patients with heart failure and chronic obstructive pulmonary disease: findings from Heart Failure and A Controlled Trial Investigating Outcomes of Exercise TraiNing (HF-ACTION). Am Heart J 2013;165(2):193-9
10. Ruikes FG, Meys AR, van de Wetering G et al. The CareWell-primary care program: design of a cluster controlled trial and process evaluation of a complex intervention targeting community-dwelling frail elderly. BMC Fam Pract 2012; 13:115
11. Fairhall N, Sherrington C, Kurrle SE et al. Effect of a multifactorial interdisciplinary intervention on mobility-related disability in frail older people: randomised controlled trial. BMC Med 2012; 10:120
12. Chou CH, Hwang CL, Wu YT. Effect of exercise on physical function, daily living activities, and quality of life in the frail older adults: a meta-analysis. Arch Phys Med Rehabil 2012;93(2):237-44
13. de Vries NM, van Ravensberg CD, Hobbelen JS et al. Effects of physical exercise therapy on mobility, physical functioning, physical activity and quality of life in community-dwelling older adults with impaired mobility, physical disability and/or multi-morbidity: a meta-analysis. Ageing Res Rev 2012;11(1):136-49
14. Szturm T, Betker AL, Moussavi Z et al. Effects of an interactive computer game exercise regimen on balance impairment in frail community-dwelling older adults: a randomized controlled trial. Phys Ther 2011;91(10):1449-62
15. Wilhelmson K, Duner A, Eklund K et al. Design of a randomized controlled study of a multi-professional and multidimensional intervention targeting frail elderly people. BMC Geriatr 2011; 11:24
16. Watt JR, Jackson K, Franz JR et al. Effect of a supervised hip flexor stretching program on gait in frail elderly patients. PM R 2011;3(4):330-5
17. Weening-Dijksterhuis E, de Greef MH, Scherder EJ et al. Frail institutionalized older persons: A comprehensive review on physical exercise, physical fitness, activities of daily living, and quality-of-life. Am J Phys Med Rehabil 2011;90(2):156-68
18. Metzelthin SF, van Rossum E, de Witte LP et al. The reduction of disability in community-dwelling frail older people: design of a two-arm cluster randomized controlled trial. BMC Public Health 2010; 10:511
19. Hagedorn DK, Holm E. Effects of traditional physical training and visual computer feedback training in frail elderly patients. A randomized intervention study. Eur J Phys Rehabil Med 2010;46(2):159-68
20. Zak M, Swine C, Grodzicki T. Combined effects of functionally-oriented exercise regimens and nutritional supplementation on both the institutionalised and free-living frail elderly (double-blind, randomised clinical trial). BMC Public Health 2009; 9:39
21. Arora VM, McGory ML, Fung CH. Quality indicators for hospitalization and surgery in vulnerable elders. J Am Geriatr Soc 2007;55 Suppl 2: S347-58
22. Chang JT, Ganz DA. Quality indicators for falls and mobility problems in vulnerable elders. J Am Geriatr Soc 2007;55 Suppl 2: S327-34
23. Groessl EJ, Kaplan RM, Rejeski WJ et al. Health-related quality of life in older adults at risk for disability. Am J Prev Med 2007;33(3):214-8
24. Alkema GE, Wilber KH, Shannon GR et al. Reduced mortality: the unexpected impact of a telephone-based care management intervention for older adults in managed care. Health Serv Res 2007;42(4):1632-50
25. Faber MJ, Bosscher RJ, Chin A et al. Effects of exercise programs on falls and mobility in frail and pre-frail older adults: A multicenter randomized controlled trial. Arch Phys Med Rehabil 2006;87(7):885-96
26. Croucher K. Making the case for retirement villages. Joseph Rowntree Foundation. Available at: https://www.jrf.org.uk/report/making-case-retirement-villages Accessed on the 28 November 2016
27. Frisoli A, Ingham SJM, Paes T.T et al. Frailty predictors and outcomes among older patients with cardiovascular disease: Data from Fragicor. Archives of Gerontology and Geriatrics 2015; 61:1-7
28. Haley MN, Wells Y.D, Holland AE. Relationship between frailty and discharge outcomes in subacute care. Australian Health Review 2014;38, 25-29
29. Heinz VH, Salzwedel A, Nosper M et al. Cardiac rehabilitation in the elderly: Outcome measurement, quality enhancement and centre comparison. European Journal of Preventive Cardiology 2012;19: S133
30. Coleman S, Cunningham C, Walsh J et al. Outcomes among older people in a post-acute inpatient rehabilitation unit, Disability and Rehabilitation: An International Multidisciplinary Journal 2012;34:15:1333-1338

Falls

1. Jeon MY, Jeong H, Petrofsky J et al. Effects of a randomized controlled recurrent fall prevention program on risk factors for falls in frail elderly living at home in rural communities. Med Sci Monit 2014; 20:2283-91
2. Giné-Garriga M, Guerra M, Unnithan VB. The effect of functional circuit training on self-reported fear of falling and health status in a group of physically frail older individuals: a randomized controlled trial. Aging Clin Exp Res 2013 ;25(3) :329-36
3. Tousignant M, Corriveau H, Roy PM et al. Efficacy of supervised Tai Chi exercises versus conventional physical therapy exercises in fall prevention for frail older adults: a randomized controlled trial. Disabil Rehabil 2013 ;35(17) :1429-35
4. Faes MC, Reelick MF, Melis RJ et al. Multifactorial fall prevention for pairs of frail community-dwelling older fallers and their informal caregivers: a dead end for complex interventions in the frailest fallers. J Am Med Dir Assoc 2011;12(6):451-8
5. Arora VM, McGory ML, Fung CH. Quality indicators for hospitalization and surgery in vulnerable elders. J Am Geriatr Soc 2007;55 Suppl 2: S347-58
6. Chang JT, Ganz DA. Quality indicators for falls and mobility problems in vulnerable elders. J Am Geriatr Soc 2007;55 Suppl 2: S327-34
7. Faber MJ, Bosscher RJ, Chin A et al. Effects of exercise programs on falls and mobility in frail and pre-frail older adults: A multicenter randomized controlled trial. Arch Phys Med Rehabil 2006;87(7):885-96
8. Thomas S, Miller M, Whitehead C et al. Falls Clinics: an opportunity to address frailty and improve health outcomes (preliminary evidence), Aging Clinical & Experimental Research 2010;22:2:170-174
9. Huang TT, Yang LH, Liu CY. Reducing the fear of falling among community-dwelling elderly adults through cognitive-behavioural strategies and intense Tai Chi exercise: A randomized controlled trial. Journal of Advanced Nursing 2011; 67:961-971
10. Hubbard RE, Story DA. Patient frailty: The elephant in the operating room. Anaesthesia 2014; 69:26-34

Minimising number of medications prescribed

1. Tjia J, Velten SJ, Parsons C et al. Studies to reduce unnecessary medication use in frail older adults: a systematic review. Drugs Aging 2013;30(5):285-307
2. Seymour MT, Thompson LC, Wasan HS et al. National Cancer Research Institute Colorectal Cancer Clinical Studies Group. Chemotherapy options in elderly and frail patients with metastatic colorectal cancer (MRC FOCUS2): an open-label, randomised factorial trial. Lancet 2011;377(9779):1749-59
3. Soni RK, Porter AC, Lash JP et al. Health-related quality of life in hypertension, chronic kidney disease, and coexistent chronic health conditions. Adv Chronic Kidney Dis 2010;17(4): e17-26
4. Shrank WH, Polinski JM, Avorn J. Quality indicators for medication use in vulnerable elders. J Am Geriatr Soc 2007;55 Suppl 2: S373-82

Good palliative care

1. Davison R, Sheerin NS. Prognosis and management of chronic kidney disease (CKD) at the end of life. Postgrad Med J 2014;90(1060):98-105
2. Lorenz KA, Rosenfeld K, Wenger N. Quality indicators for palliative and end-of-life care in vulnerable elders. J Am Geriatr Soc 2007;55 Suppl 2: S318-26
3. Waldrop DP. Treatment at the end of life. Journal of Gerontological Social Work 2008;50 (S1): 267-292
4. Heyland D, Dodek P, Mehta S et al. Admission of the very elderly to the intensive care unit: Family members’ perspectives on clinical decision-making from a multicenter cohort study Palliative Medicine 2005;29:4:324-335
5. Finucane A, Stevenson B, Moyes R et al. Improving end-of-life care in nursing homes: Implementation and evaluation of an intervention to sustain quality of care, Palliative Medicine 2013;27:8:772-778
6. Andersson M, Hallberg I, Edberg A. Old people receiving municipal care, their experiences of what constitutes a good life in the last phase of life: A qualitative study, International Journal of Nursing Studies 2008;45:6:818-828
7. Groninger H, Childress M. Samuel Beckett's 'Rockaby': Dramatizing the plight of the solitary elderly at life's end, Perspectives in Biology and Medicine 2007;50:2:260-275

Good vision

1. Rowe S, MacLean CH. Quality indicators for the care of vision impairment in vulnerable elders. J Am Geriatr Soc 2007;55 Suppl 2: S450-6

Continence

1. Fung CH, Spencer B, Eslami M et al. Quality indicators for the screening and care of urinary incontinence in vulnerable elders. J Am Geriatr Soc 2007;55 Suppl 2: S443-9
2. Fusco D, Bochicchio G, Onder G et al. Predictors of rehabilitation outcome among frail elderly patients living in the community. Journal of The American Medical Directors Association 2009; 10:5:335-341

Good nutritional status

1. Lammes E, Rydwik E, Akner G. Effects of nutritional intervention and physical training on energy intake, resting metabolic rate and body composition in frail elderly. a randomised, controlled pilot study. J Nutr Health Aging 2012;16(2):162-7
2. Rydwik E, Frändin K, Akner G. Effects of a physical training and nutritional intervention program in frail elderly people regarding habitual physical activity level and activities of daily living--a randomized controlled pilot study. Arch Gerontol Geriatr 2010;51(3):283-9
3. Zak M, Swine C, Grodzicki T. Combined effects of functionally-oriented exercise regimens and nutritional supplementation on both the institutionalised and free-living frail elderly (double-blind, randomised clinical trial). BMC Public Health 2009; 9:39
4. Reuben DB. Quality indicators for the care of undernutrition in vulnerable elders. J Am Geriatr Soc 2007;55 Suppl 2: S438-42
5. Bates-Jensen BM, MacLean CH. Quality indicators for the care of pressure ulcers in vulnerable elders. J Am Geriatr Soc 2007;55 Suppl 2: S409-16
6. Hansen T, Lambert H, Faber J. Ingestive Skill Difficulties are Frequent Among Acutely-Hospitalized Frail Elderly Patients, and Predict Hospital Outcomes, Physical & Occupational Therapy in Geriatrics 2012;30:4:271-287
7. Naharci MI. Is weight loss an outcome of or a contributory factor for vascular dementia in frail elderly people?. Medical Principles and Practice 2015; 24:298

Pain

1. Etzioni S, Chodosh J, Ferrell BA et al. Quality indicators for pain management in vulnerable elders. J Am Geriatr Soc 2007;55 Suppl 2: S403-8
2. Lorenz KA, Rosenfeld K, Wenger N. Quality indicators for palliative and end-of-life care in vulnerable elders. J Am Geriatr Soc 2007;55 Suppl 2: S318-26
3. Yoon E, Doherty JB. Arthritis pain. Journal of Gerontological Social Work 2008; 50:79-103

Mental and psychological health

Maintaining cognition

1. Clegg A, Siddiqi N, Heaven A et al. Interventions for preventing delirium in older people in institutional long-term care. Cochrane Database of Systematic Reviews 2014;1: CD009537
2. Hubbard RE, Story DA. Patient frailty: The elephant in the operating room. Anaesthesia 2014; 69:26-34
3. Fusco D, Bochicchio G, Onder G et al. Predictors of rehabilitation outcome among frail elderly patients living in the community, Journal of The American Medical Directors Association 2009;10:5:335-341
4. Netten A, Darton R, Bäumker T et al. Improving housing with care choices for older people: an evaluation of extra care housing. London: Housing Learning and Improvement Network; Personal Social Services Research Unit 2011. Available at: http://www.housinglin.org.uk/_library/Resources/Housing/Research_evaluation/PSSRUsummary.pdf Accessed on the 27 November 2016
5. de Rooij SE, Abu-Hanna A, Levi M et al. Factors that predict outcome of intensive care treatment in very elderly patients: a review. Crit Care 2005;9(4): R307-14
6. Arora VM, McGory ML, Fung CH. Quality indicators for hospitalization and surgery in vulnerable elders. J Am Geriatr Soc 2007;55 Suppl 2: S347-58
7. O'Caoimh R, Gao Y, Svendrovski A, Healy E, O'Connell E, O'Keeffe G, Cronin U, O'Herlihy E, Cornally N, Molloy WD. Screening for markers of frailty and perceived risk of adverse outcomes using the Risk Instrument for Screening in the Community (RISC). BMC Geriatr 2014; 14:104

Experience measures

Treated with respect and dignity

1. Muntinga ME, Hoogendijk EO, van Leeuwen KM et al. Implementing the chronic care model for frail older adults in the Netherlands: study protocol of ACT (frail older adults: care in transition). BMC Geriatr 2012; 12:19
2. Improvement and Development Agency. Why bother? improving the quality of life for older people. Available at: http://www.scie-socialcareonline.org.uk/why-bother-improving-the-quality-of-life-for-older-people/r/a11G00000017zHbIAIAccessed on the 28 November 2016
3. Owen T, Meyer J, Cornell M et al. My home life: promoting quality of life in care homes: report. Joseph Rowntree Foundation. Available at: <https://www.jrf.org.uk/file/42952/download?token=L-_tt7DX&filetype=full-report> Accessed on the 28 November 2016
4. Bolton J. Wiltshire Council: help to live at home service: an outcome-based approach to social care: a case study report. Oxford Brookes University. Institute of Public Care. Available at: <https://ipc.brookes.ac.uk/publications/Wiltshire_Council_Help_to_Live_at_Home_IPC_Report_April_2012.pdf> Accessed on the 28 November 2016
5. Andersson M, Hallberg I, Edberg A. Old people receiving municipal care, their experiences of what constitutes a good life in the last phase of life: A qualitative study. International Journal of Nursing Studies 2008; 45:6:818-828

Involved in discussions and decisions

1. Muntinga ME, Hoogendijk EO, van Leeuwen KM et al. Implementing the chronic care model for frail older adults in the Netherlands: study protocol of ACT (frail older adults: care in transition). BMC Geriatr 2012; 12:19
2. Mutasingwa DR, Ge H, Upshur RE. How applicable are clinical practice guidelines to elderly patients with comorbidities? Can Fam Physician 2011;57(7): e253-62
3. de Rooij SE, Abu-Hanna A, Levi M et al. Factors that predict outcome of intensive care treatment in very elderly patients: a review. Crit Care 2005;9(4): R307-14.
4. Roe, B, Beech R, Harris M et al. Improving quality of life for older people in the community: findings from a local Partnerships for Older People Project innovation and evaluation. Prim Health Care Res Dev, 2011;12(3), 200-213.
5. Callaghan L, Towers A. Feeling in control: comparing older people's experiences in different care settings. Ageing and Society 2014;34: 1427-1451
6. Improvement and Development Agency. Why bother? improving the quality of life for older people. Available at: <http://www.scie-socialcareonline.org.uk/why-bother-improving-the-quality-of-life-for-older-people/r/a11G00000017zHbIAI> Accessed on the 28 November 2016
7. Owen T, Meyer J, Cornell M et al. My home life: promoting quality of life in care homes: report. Joseph Rowntree Foundation. Available at: <https://www.jrf.org.uk/file/42952/download?token=L-_tt7DX&filetype=full-report> Accessed on the 28 November 2016
8. Bolton J. Wiltshire Council: help to live at home service: an outcome-based approach to social care: a case study report. Oxford Brookes University. Institute of Public Care. Available at: <https://ipc.brookes.ac.uk/publications/Wiltshire_Council_Help_to_Live_at_Home_IPC_Report_April_2012.pdf> Accessed on the 28 November 2016
9. Social Care Institute for Excellence. Improving care packages and outcomes of older people. Community Care 2008;32-33
10. Croucher K. Making the case for retirement villages. Joseph Rowntree Foundation. Available at: <https://www.jrf.org.uk/report/making-case-retirement-villages> Accessed on the 28 November 2016
11. Laforest S, Nour K, Gignac MA et al. The role of social reinforcement in the maintenance of short-term effects after a self-management intervention for frail housebound seniors with arthritis. Can J Aging 2012;31(2):195-207

Reduced anxiety and worry about healthcare

1. Behm L, Wilhelmson K, Falk K et al. Positive health outcomes following health-promoting and disease-preventive interventions for independent very old persons: long-term results of the three-armed RCT Elderly Persons in the Risk Zone. Arch Gerontol Geriatr 2014;58(3):376-83
2. Rizzoli R, Reginster JY, Arnal JF et al. Quality of life in sarcopenia and frailty. Calcif Tissue Int 2013;93(2):101-20
3. Giné-Garriga M, Guerra M, Unnithan VB. The effect of functional circuit training on self-reported fear of falling and health status in a group of physically frail older individuals: a randomized controlled trial. Aging Clin Exp Res 2013;25(3):329-36
4. Ruikes FG, Meys AR, van de Wetering G et al. The CareWell-primary care program: design of a cluster controlled trial and process evaluation of a complex intervention targeting community-dwelling frail elderly. BMC Fam Pract 2012 ;13 :115
5. van Mourik Y, Moons KG, Bertens LC et al. Triage of frail elderly with reduced exercise tolerance in primary care (TREE). A clustered randomized diagnostic study. BMC Public Health 2012; 12:385
6. Melis RJ, van Eijken MI, van Achterberg T et al. The effect on caregiver burden of a problem-based home visiting programme for frail older people. Age Ageing 2009;38(5):542-7
7. Netten A, Darton R, Bäumker T et al. Improving housing with care choices for older people: an evaluation of extra care housing. London: Housing Learning and Improvement Network; Personal Social Services Research Unit 2011. Available at: http://www.housinglin.org.uk/_library/Resources/Housing/Research_evaluation/PSSRUsummary.pdf Accessed on the 27 November 2016
8. Prior MK, Bahret BA, Pasupuleti S. The efficacy of a senior outreach program in the reduction of hospital readmissions and emergency department visits among chronically ill seniors. Social Work in Health Care 2012; 51: 345-360
9. Huang TT, Yang L.-H, Liu CY. Reducing the fear of falling among community-dwelling elderly adults through cognitive-behavioural strategies and intense Tai Chi exercise: A randomized controlled trial. Journal of Advanced Nursing 2011; 67:961-971
10. Schulze T, Maercker A, Horn AB. Mental health and multimorbidity: psychosocial adjustment as an important process for quality of life. Gerontology 2014;60(3):249-54
